# New insights into the cortex-to-stele ratio show it to effectively indicate inter- and intraspecific function in the absorptive roots of temperate trees

**DOI:** 10.3389/fpls.2023.1061503

**Published:** 2023-01-20

**Authors:** Xiangjuan Liu, Yanjun Du, Yin Ren, Siyuan Wang, Yan Wang, Zhongyue Li, Wenna Wang

**Affiliations:** ^1^ Key Laboratory of Germplasm Resources of Tropical Special Ornamental Plants of Hainan Province, College of Forestry, Hainan University, Haikou, China; ^2^ School of Forestry, Northeast Forestry University, Harbin, China; ^3^ Taishan Forest Ecosystem Research Station of State Forestry Administration, State Forestry and Grassland Administration Key Laboratory of Silviculture in downstream areas of the Yellow River, College of Forestry, Shandong Agricultural University, Taian, China

**Keywords:** cortex-to-stele ratio, branch order, root anatomy, morphology and chemistry, resource acquisition strategy

## Abstract

The cortex-to-stele ratio (CSR), as it increases from thin- to thick-root species in angiosperms, is theorised to effectively reflect a compensation for the ‘lag’ of absorption behind transportation. But it is still not known if this compensatory effect exists in gymnosperm species or governs root structure and function within species. Here, anatomical, morphological, and tissue chemical traits of absorptive roots were measured in three temperate angiosperm and three gymnosperm species. Differences in the CSR and the above functional traits, as well as their intraspecific associations, were analyzed and then compared between angiosperms and gymnosperms. At the intraspecific level, the CSR decreased with increasing root order for all species. The expected functional indication of the CSR was consistent with decreases in specific root length (SRL) and N concentration and increases in the C to N ratio (C:N ratio) and the number of and total cross-sectional area of conduits with increasing root order, demonstrating that the CSR indicates the strength of absorption and transportation at the intraspecific level, but intraspecific changes are due to root development rather than the compensatory effect. These trends resulted in significant intraspecific associations between the CSR and SRL (*R* ^2^ = 0.36 ~ 0.80), N concentration (*R* ^2^ = 0.48 ~ 0.93), the C:N ratio (*R* ^2^ = 0.47 ~ 0.91), and the number of (*R* ^2^ = 0.21 ~ 0.78) and total cross-sectional area (*R* ^2^ = 0.29 ~ 0.72) of conduits in each species (*p*< 0.05). The overall mean CSR of absorptive roots in angiosperms was four times greater than in gymnosperms, and in angiosperms, the CSR was significantly higher in thick- than in thin-rooted species, whereas in gymnosperms, the interspecific differences were not significant (*p* > 0.05). This suggests that the compensation for the lag of absorption *via* cortex thickness regulation was stronger in three angiosperm species than in three gymnosperm species. In addition, there was poor concordance between angiosperms and gymnosperms in the relationships between CSRs and anatomical, morphological, and tissue chemical traits. However, these gymnosperm species show a more stable intraspecific functional association compared to three angiosperm species. In general, absorptive root CSRs could manifest complex strategies in resource acquisition for trees at both intra- and interspecific levels.

## 1 Introduction

Absorptive roots, the primary structures absorbing water and nutrients from the soil, are major participants and contributors in C and nutrient cycles of terrestrial ecosystems (Pregitzer et al., 2002; Wells and Eissenstat, 2003; Guo et al., 2008a; McCormack et al., 2012). The cortex and stele (*i.e.*, the vascular cylinder) of absorptive roots are important tissues for the uptake and transport, respectively, of water and nutrients (Esau, 1977; Peterson et al., 1999; Lux et al., 2004). However, there is a nonlinear relationship (*i.e.*, an allometric relationship) between the cortex and stele at the interspecific level (Kong et al., 2017). In other words, the ratio of cortex thickness to stele radius varies disproportionately between species. The allometric relationships arise from an optimally functional balance between nutrient absorption and transportation in absorptive roots (Kong et al., 2017). The cortex-to-stele ratio (CSR) of absorptive roots has thus been proposed as an effective indicator for evaluating the balance between resource absorption *via* the cortex and transportation *via* conduits (vessels in angiosperms and tracheids in gymnosperms, Kong et al., 2017; Wang et al., 2018). Therefore, the study of the variation of the CSR and the associated relative changes to absorption and transport is of great significance for fully uncovering the mechanism underlying the relationship between root structure and function, as well as further understanding how the resource acquisition strategies of tree absorptive roots regulates the functioning of the forest system (Guo et al., 2008b; Wang et al., 2018).

Previous studies on the CSR in absorptive roots have mainly focused on the interspecific level (Kong et al., 2014; Wang et al., 2018), ignoring the intraspecific level. Studies on the interspecific level have primarily concentrated on angiosperms and found a compensatory effect between absorption and transportation: as the transportation increases with roots becoming thicker from one species to another, its absorption increases relatively greater (Guo et al., 2008b; Kong et al., 2017; Wang et al., 2018). In theory, because transportation of absorptive roots is proportional to the fourth power of stele diameter (Tyree and Ewers, 1991) and absorption to the second power of cortex thickness (Kong et al., 2017), nutrient absorption will ‘lag’ far behind transportation as root diameter gets infinitely thicker. However, the absorption and transportation of absorptive roots are actually in dynamic equilibrium. This is driven by the allometric relationship between cortex and stele across thin- and thick-root species (Kong et al., 2017). As average absorptive root thickness progresses from thin to thick, the thickness of the cortex tends to increase relative to the radius of the stele to reduce the nutrient absorption lag (Kong et al., 2014; Valverde-Barrantes et al., 2016; Kong et al., 2017). Put simply, tree species with thicker roots have a greater CSR. Conversely, at the intraspecific level, where thickness is affected by root development, stele radius increases whereas cortex thickness decreases, or even abscises, due to lignification and suberification with increasing root order (Jia et al., 2013; Gu et al., 2014). Variations of cortex thickness and stele radius at the intraspecific level do not conform to the functional balance found at the interspecific level. Therefore, we proposed the first hypothesis that the compensatory effect indicated by CSR at the interspecific level does not exist at the intraspecific level. Nevertheless, CSR should still indicate the strength of absorption versus transportation at the intraspecific level, where CSR is determined by root development, but this has not yet been investigated.

Based on the functional indication of CSR at the intraspecific level, and to more fully understand the regulatory mechanisms relating absorptive root structure to function, an analysis of the relationship between CSR and vital functional traits at the intraspecific level is required. Vital functional traits of absorptive roots include a set of anatomical, morphological, and tissue chemical traits (Pregitzer et al., 2002; Ma et al., 2018; Lynch et al., 2021; Wang et al., 2022). However, which, if any, of these functional traits closely correlate with CSR at the intraspecific level is unclear. Previous studies have found that functional traits differed in the degree to which they respond to environmental changes (Pregitzer et al., 2000; Ostonen et al., 2007; Li et al., 2015). For absorptive root tissue chemical traits, for instance, N concentration and C:N ratio are more sensitive functional traits in response to environmental changes than C concentration (Terzaghi et al., 2013; Wang et al., 2022). In addition, different types of anatomical traits, *e.g.*, qualitative and quantitative traits, have shown differing degrees of effects on absorptive root growth and physiology. For example, previous studies found that larger diameter cortical cells and conduits could reduce metabolic cost and enhance vertical transport efficiency, respectively, to a greater extent than did greater numbers of cortical cells and conduits (Tyree and Ewers, 1991; Chimungu et al., 2014; Chimungu et al., 2014b). But, larger diameter cortical cells and conduits may also be disadvantageous for tree fitness by reducing water conductance (Huang and Eissenstat, 2000) and being more vulnerable to embolisms (Ewers et al., 1990; Hacke et al., 2000). Given these differences between vital functional traits in both their responses to environmental change and their influences on tree growth and physiology, we hypothesized that distinct differences were also exist in the association degrees between them and CSR. Thus, studying the associations of functional traits with CSR is important for understanding the resource acquisition strategies of absorptive roots in trees.

Between thin- and thick-root species, the allometric relationship of cortex and stele has been proposed in angiosperms, but studies on gymnosperms are still scarce (Kong et al., 2017). Compared with angiosperms, absorptive roots of gymnosperms have substantially weaker volumetric flow rates, a thinner cortex, and (in the family Pinaceae) higher ectomycorrhizal colonization (Sperry et al., 2006; Guo et al., 2008b). We therefore hypothesized that such differences may contribute to disparities between angiosperms and gymnosperms in vertical transport efficiency and the reliance on mycorrhizas for resource acquisition, which may reflect as different relationships between absorptive root structure (*e.g.*, CSR) and function and different associations between vital traits and CSR. In this context, studying the mechanisms underlying the differences in the functional relationship and trait associations of CSR between angiosperms and gymnosperms will lead to a better understanding of the resource acquisition strategies of absorptive roots at the level of forest stands.

**Figure 1:**
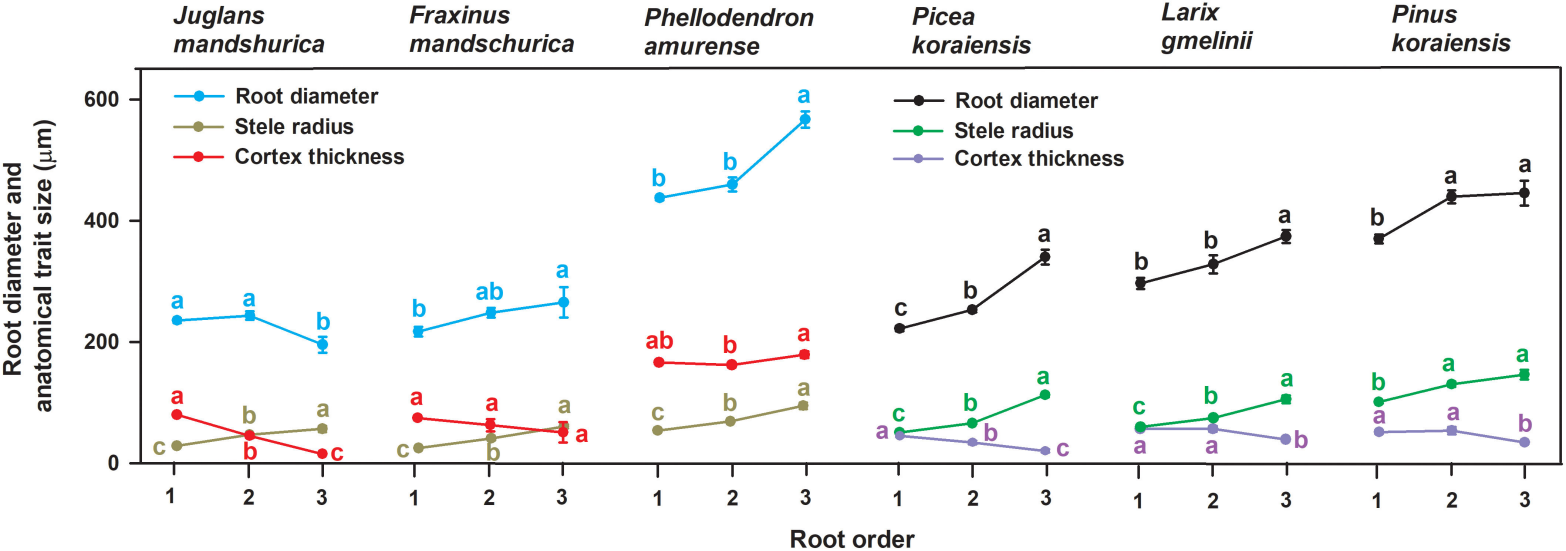
Variations of root diameter, stele radius and unilateral cortex thickness among the first three absorptive root orders within three angiosperms and three gymnosperms tree species (*n* = 30). For each trait, different lower-case letters indicate significant differences (*p*< 0.05) among root orders within species.

In this study, we chose six temperate tree species, including three broad-leaved tree species—*Juglans mandschurica* Maxim., *Fraxinus mandschurica* Rupr., *Phellodendron amurense* Rupr.—and three conifer tree species—*Picea koraiensis* Nakai., *Larix gmelinii* Rupr. and *Pinus koraiensis* Sieb.—as the experimental plants. These tree species cover a range of average absorptive root tip diameters from 224 μm to 487 μm (**Figure 1**). The measurement of anatomical, morphological, and tissue chemical traits were performed for the first three order roots based on the root order system (the distal roots numbered as first-order roots, Pregitzer et al., 2002). After the calculation of CSR (*i.e.*, the ratio of cortex thickness to stele radius), the intraspecific relationships between CSR and anatomical, morphological, and tissue chemical traits were determined, and differences in these relationships and in the interspecific variation of CSR were compared between angiosperms and gymnosperms. The aim of this study was to better understand the functional indications of CSR at both the intra- and interspecific levels and thus more broadly understand the differences and similarities in resource acquisition strategies of tree absorptive roots.

## 2 Materials and methods

### 2.1 Study site and tree species chosen

This study was performed within the Maoershan Forest Research Station (127°30′~127°34′E,45°21′~45°25′N) in northeast China. The region has a warm temperate monsoon climate. The mean annual temperature is 2.8°C, with a mean monthly maximum of 20.9°C (July) and a mean monthly minimum of -19.6°C (January). The frost-free season lasts 120–140 d throughout the year, with an average accumulated degree days above 10°C of 2526°C. The annual precipitation is 723 mm, and the rainfall is mainly concentrated in July and August, accounting for 31.2% and 21.1% of annual precipitation, respectively. The annual evaporation is 1094 mm. The study site is in a valley with an elevation of 300 m and a 10°–15° slope. Soils are well-drained Hap-Boric Luvisols with high organic matter content (Wang et al., 2006).

In this study, six afforested, pure plantations, each containing a different tree species, were selected, species included *J. mandshurica*, *F. mandschurica*, *P. amurense*, *L. gmelinii*, *Pinus koraiensis*, and *Picea koraiensis*. All plantations were 35 years old, with 2.0×1.5 m interspacing, and located on the upper part of the hillside. In each, six 4×4 m sampling plots were established (six plots per species). The total area of each plantation was over five hectares.

### 2.2 Root sample collection

Root segments of each tree species were sampled in July 2019. In each sampling plot, three sampling points were randomly located and the upper 20 cm layer of soil and the litter were gently removed in a 50 cm by 50 cm area. Then root branches, including the intact first five-order roots, were found along the main root of the target tree species and cut by garden scissors. Root segments from the three sampling points were combined. After being washed with deionised water, root samples from the same site were divided randomly into two groups. One group was rapidly plunged into a formalin-aceto-alcohol (FAA) solution (90ml of 50% ethanol, 5ml of 100% glacial acetic acid, and 5ml of 37% formaldehyde) for further anatomical analysis and another group was put into a Ziploc bag for subsequent morphological and tissue chemical analyses. All root samples were kept cool before transport to the laboratory in a cooler with ice packs inside. Root samples in FAA and in Ziploc bags were then conserved at 4°C and -10°C, respectively, for subsequent functional trait measurement.

### 2.3 Root anatomy

For each sampling plot, five individual roots from each root order were chosen randomly from the FAA solution. Thus 30 individual roots (*i.e*., five individual roots from each of the six sampling plots) per root order were used for the measurement of anatomical traits per species. Following the methods of Guo et al. (2008b) and Gu et al. (2014), these individual roots underwent multiple chemical treatment steps (*e.g.*, dehydration and dealcoholization) before they were embedded in paraffin. Slides of 8-μm-thick root sections were prepared with a microtome and stained with safranin-fast green (2%). Images of each cross-section were photographed under a microscope (BX-51, Olympus Corporation, Tokyo, Japan), and anatomical structures including cortex thickness, stele radius, mean conduit diameter, and mean diameter of cortical cells were measured using Motic Images Advanced 3.2 (Motic Corporation, Zhejiang, China). Numbers of conduits per stele and cortical cell layers were also recorded concurrently. The CSR was calculated by dividing cortex thickness with stele radius for each root order within each species. The total cross-sectional area of conduits was calculated using each conduit’s radius based on the assumption that each conduit was a circle. A cross-sectional area of the cortex was calculated as the cross-sectional area of root minus that of stele based on the assumption that the cross sections of root and stele were both circles. The specific conductivity (*K*_s_) was calculated using Hagen–Poiseuille’s law (Tyree and Ewers, 1991):


$$K_{s}=\;(\pi \rho /128\eta Aw)\;\sum_{i=1}^{n}\left(d_{i}^{4}\right)$$

where *K*_s_ is theoretical axial conductivity along a root tip, ρ is the density of water (where temperature was set at 18°C, consistent with the root respiration measurement), η is the dynamic viscosity, *Aw* is the area of the stele, *d* is the diameter of the *i*th conduit, and *n* is the number of conduits in the xylem.

For each anatomical trait, there were six replicates per root order per species: a mean trait value calculated as the average of the five individual roots from the same sampling plot.

### 2.4 Root morphological and tissue chemical traits

For each sampling plot, 100–200 individual roots from each root order were selected for morphological analysis. Individual roots were scanned using Expression 10000XL 1.0 scanner (Epson Telford Ltd, Telford, UK). Then, the root image was analyzed using WinRhizo (2004b, Regent Instruments, Inc., Québec, Canada) to acquire a set of data including root diameter, length, and volume. All the scanned roots were packaged into paper bags to dry at 65°C until constant weight and weighed (with 0.0001g precision). SRL was calculated as the ratio of total root length to root dry weight (m/g) and tissue density as the ratio of root dry weight to root volume (g/cm^3^).

The remaining 500–1000 roots from each sampling plot were divided based on root order and dried until constant weight in an oven at 65°C and weighed (with 0.0001g precision) then ground to a fine powder. Tissue total C and total N concentrations were measured using an elemental analyser (Vario Macro, Elementar Co, Germany), and the C:N ratio was calculated as the ratio of total C concentration to total N concentration. This resulted in six replicates of each morphological and tissue chemical trait per root order for each species.

### 2.5 Data analysis

For each tree species, the mean and standard error of each root anatomical, morphological, and tissue chemical trait from each order root was calculated using six replicates. LSD multiple comparison tests were performed to statistically test differences between the functional traits of tree species and root orders. A group-wise regression was used to test the intraspecific relationships between CSR and anatomical, morphological, and tissue chemical traits using tree species as groups: between the CSR and (1) the anatomical traits of the mean conduit diameter, number of conduits per stele, total cross-sectional area of conduits, mean diameter of cortical cells, number of cortical cell layers, and cross-sectional area of the cortex; (2) the morphological traits of root diameter, SRL, and tissue density; (3) the chemical traits of tissue total N concentration, total C concentration, and C:N ratio. The correlation coefficients (Adj *R* ^2^), its standard error, significance level and *df* performed by group-wise regression analysis were reported. According to the best fit of Standardized major axis (SMA) regression analysis for estimating the underlying allometric relationships used in ecology (Warton et al., 2012; Michaletz et al., 2014), the interspecific difference of the significance level in the slopes of regression models were tested by SMA regression. In addition, intraspecific correlation coefficients (*R* ^2^), *p* value, y-intercept and slope for each species examined by SMA regression were recorded (**Tables S1–4**). The above data (*i.e.*, CSR and anatomical, morphological, and tissue chemical traits) were log-transformed prior to SMA analysis. In addition, the interrelationships (*n* = 6, *i.e*., six species) between CSR and multiple anatomical, morphological, and tissue chemical traits were analyzed by principal component analysis (PCA).

Because the first three orders of roots are generally considered as a functional module (*i.e*., absorptive roots, Pregitzer et al., 2002; Guo et al., 2008b), reference to the experimental design of Li et al. (2021), we used the first three orders of roots as measurement unit in the current study. That is, for each species, the above intraspecific relationships were based on 18 replicates per species (*i.e.*, six replicates from each of three root orders).

Group-wise regression and LSD multiple comparison tests were performed using SPSS for Windows version 22.0 (IBM Corp., Armonk, NY, United States). SMA regression analyses were performed using SMATR (Version 2.0, http://www.bio.mq.edu.au/ecology/SMATR/, accessed on 29 July 2006, Falster et al., 2006). PCA was conducted in the CANOCO software (CANOCO V. 4.5).

## 3 Results

### 3.1 Intraspecific differences of CSRs and anatomical, morphological, and tissue chemical traits of absorptive roots

**Figure 2:**
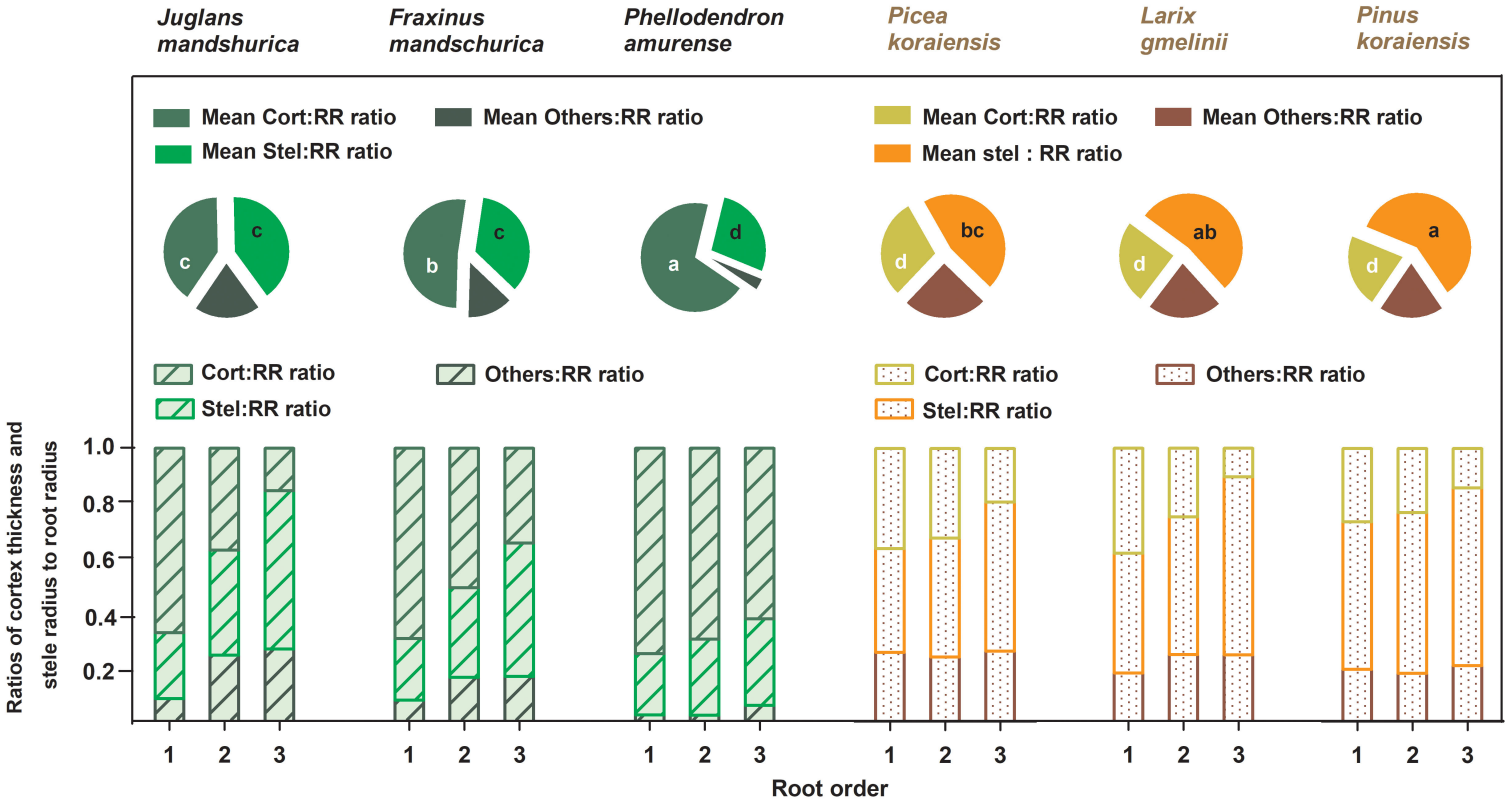
Ratios of unilateral cortex thickness (Cort : RR ratio), stele radius (Stel : RR ratio) and unilateral cross-sectional length of the other components (Others : RR ratio) to root radius for the first three absorptive root orders within three angiosperms and three gymnosperms tree species (*n* = 30). For each order root per species, mean ratios of unilateral cortex thickness (Mean Cort : RR ratio), stele radius (Mean Stel : RR ratio) and unilateral cross-sectional length of the other components (Mean Others : RR ratio) to root radius were calculated across root orders, respectively (*n* = 30). Different white and black lower-case letters on the pie chart indicate significant differences (*p*< 0.05) in Mean Cort : RR ratio and Mean Stel : RR ratio among six species, respectively.

**Figure 3:**
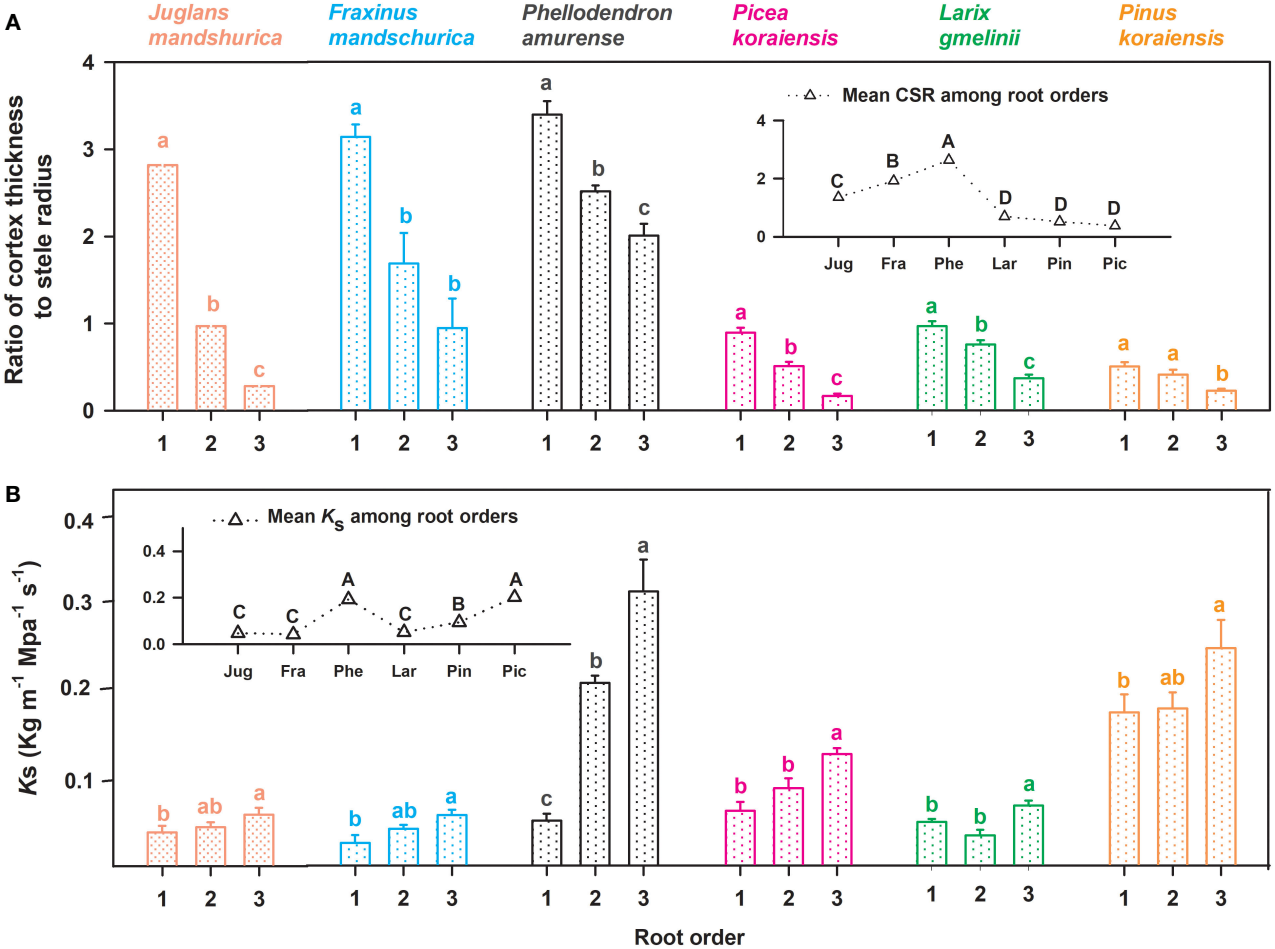
Ratio of unilateral cortex thickness to stele radius (CSR, **(A)** and the specific conductivity (*K*_s_, **(B)** for the first three absorptive root orders in three angiosperms and three gymnosperms tree species (*n* = 30). For each trait, in the subplots the mean value was calculated among root orders at the intraspecific level. Different lower and upper case letters indicate significant differences (*p*< 0.05) among root orders within each species and among six species, respectively.

No matter angiosperm or gymnosperm species, absorptive root cortex thickness generally decreased (except for *P. amurense*) while stele radius and root diameter (except for *J. mandshurica*) increased with increasing root order within each tree species (**Figure 1**). The ratios of cortex thickness and stele radius to root radius had similar intraspecific variations to those of cortex thickness and stele radius, respectively (**Figure 2**). The intraspecific variation of cortex thickness and stele radius led to an obvious decrease of CSRs with increasing root order, with significant differences between the first and the third order roots of every species (*p*< 0.05, **Figure 3A**).

For conduit-related traits, the mean diameter (except for *J. mandshurica* and *Pinus koraiensis*), number, total cross-sectional area of conduits, and *K*_s_ all increased with increasing root order, with significant differences between the first and the third root orders within each species (**Table 1** and **Figure 3B**). Differences between the means of root orders, as a percent of the overall mean of the species, were much greater for the number of conduits (28.2%–90.2%) than for the mean conduit diameters (4.2%–12.9%). For cortical cell traits, the intraspecific variations of their number and mean diameter were more dependent on tree species and the specific trait (**Table 2**). Specifically, the number of cortical cell layers changed little in *F. mandschurica* and *P. amurense* (*p* > 0.05) but obviously decreased with root order in the other tree species. However, the mean diameter of cortical cells varied little among root orders for most species (*p* > 0.05, except for *J. mandshurica*) (**Table 2**).

**Table 1:** The mean and standard error of conduit traits for the first three absorptive root orders in three angiosperms and three gymnosperms tree species (*n* = 30).

Anatomical traits	Mean conduit diameter (μm)			Number of conduits per stele			Total cross-sectional area of conduits (μm^2^)		
*df*	29	29	29	29	29	29	29	29	29
Root order	1	2	3	1	2	3	1	2	3
*Juglans mandshurica*	6.0 ± 0.1 a	5.6 ± 0.2 a	5.9 ± 0.2 a	9.8 ± 0.2 c	44.0 ± 6.3 b	62.3 ± 6.2 a	278.3 ± 15.6 c	1040.1 ± 125.5 b	1777.9 ± 252.3 a
*Fraxinus mandschurica*	5.2 ± 0.4 b	5.6 ± 0.2 ab	6.1 ± 0.2 a	10.3 ± 0.8 c	38.7 ± 4.0 b	94.8 ± 13.1 a	216.3 ± 28.7 c	944.1 ± 101.7 b	2718.2 ± 276.8 a
*Phellodendron amurense*	7.2 ± 0.3 b	8.9 ± 0.1 ab	9.3 ± 0.2 a	18.3 ± 0.8 b	45.3 ± 3.0 b	129.6 ± 26.7 a	761.9 ± 67.5 b	2823.9 ± 152.4 b	8658.5 ± 1612.0 a
*Picea koraiensis*	7.4 ± 0.2 b	7.5 ± 0.1 b	9.2 ± 0.3 a	11.5 ± 0.7 b	14.2 ± 0.8 b	41.4 ± 3.3 a	496.4 ± 26.1 b	635.1 ± 56.4 b	2708.9 ± 211.5 a
*Larix gmelinii*	8.0 ± 0.2 c	9.0 ± 0.2 b	9.8 ± 0.3 a	15.2 ± 0.9 c	23.4 ± 2.3 b	46.5 ± 3.2 a	770.2 ± 57.3 b	1487.0 ± 199.0 b	3603.2 ± 426.2 a
*Pinus koraiensis*	12.1 ± 0.7 ab	11.9 ± 0.1 b	13.1 ± 0.2 a	24.9 ± 1.0 c	33.8 ± 2.3 b	44.3 ± 2.5 a	2959.9 ± 482.5 b	3736.5 ± 228.8 b	5985.2 ± 367.3 a
*df*	1	1	1	1	1	1	1	1	1
Angiosperms	6.1 ± 0.6 B	6.7 ± 1.0 B	7.1 ± 1.0 B	12.8 ± 2.5 B	42.7 ± 6.4 A	95.6 ± 28.4 A	418.8 ± 155.9 B	1602.7 ± 541.0 A	4384.8 ± 2210.5 A
Gymnosperms	9.2 ± 1.3 A	9.5 ± 1.1 A	10.7 ± 1.1 A	17.2 ± 3.6 A	23.8 ± 5.4 B	44.1 ± 4.2 B	1408.8 ± 753.8 A	1952.9 ± 812.4 A	4099.1 ± 942.0 A

For each trait, data present in the bottom two rows are means ± SE among three tree species for each life form. Different lower and upper case letters indicate significant differences (p< 0.05) among root orders within each species and between life forms, respectively.

**Table 2:** The mean and standard error of cortical cell traits for the first three absorptive root orders in three angiosperms and three gymnosperms tree species (*n* = 30).

	Mean diameter of cortical cell (μm)			Number of cortical cell layers			Cross-sectional area of cortex (μm^2^)		
Root order	1	2	3	1	2	3	1	2	3
*df*	29	29	29	29	29	29	29	29	29
*Juglans mandshurica*	12.7 ± 0.1 a	11.5 ± 0.8 ab	10.4 ± 0.4 b	6.6 ± 0.1 a	4.3 ± 0.3 b	2.3 ± 0.2 c	34188.8 ± 1278.8 a	20005.7 ± 2086.8 b	6228.8 ± 796.5 c
*Fraxinus mandschurica*	11.9 ± 0.3 a	12.3 ± 0.5 a	11.7 ± 0.3 a	6.5 ± 0.1 a	5.4 ± 0.7 a	4.4 ± 1.0 a	29751.9 ± 2244.8 a	30081.9 ± 5801.1 a	30970.5 ± 11110.3 a
*Phellodendron amurense*	20.5 ± 0.5 a	20.9 ± 1.1 a	22.4 ± 0.9 a	8.1 ± 0.1 a	8.1 ± 0.2 a	8.1 ± 0.5 a	135744.7 ± 4108.0 b	146155.3 ± 7521.9 b	199552.1 ± 11461.5 a
*Picea koraiensis*	16.5 ± 0.5 a	16.7 ± 0.3 a	14.0 ± 1.9 a	2.6 ± 0.3 a	2.3 ± 0.3 a	1.5 ± 0.2 b	18559.0 ± 1223.2 a	15829.6 ± 1798.7 a	13573.0 ± 2516 a
*Larix gmelinii*	17.4 ± 0.8 a	17.0 ± 0.6 a	17.4 ± 1.1 a	3.4 ± 0.2 a	3.2 ± 0.2 a	2.0 ± 0.1 b	28040.4 ± 1990.3 a	33657.3 ± 4367.2 a	27160.4 ± 2392 a
*Pinus koraiensis*	18.3 ± 0.4 a	18.8 ± 1.5 a	17.3 ± 2.1 a	3.1 ± 0.2 a	2.7 ± 0.2 a	2.1 ± 0.2 b	38464.9 ± 2625.2 ab	49918.6 ± 6221.1 a	32247.5 ± 3128.2 b
*df*	1	1	1	1	1	1	1	1	1
Angiosperms	15.1 ± 2.4 B	14.9 ± 2.8 B	14.8 ± 3.3 A	7.0 ± 0.9 A	5.9 ± 1.4 A	5.0 ± 2.5 A	66561.7 ± 155.9 A	65414.2 ± 541.0 A	78917.1 ± 2210.5 A
Gymnosperms	17.4 ± 0.4 A	17.5 ± 1.1 A	16.2 ± 1.7 A	3.0 ± 0.3 B	2.7 ± 0.4 B	1.9 ± 0.3 B	28354.8 ± 753.8 B	33135.2 ± 812.4 B	24326.9 ± 942.0 B

For each trait, data present in the bottom two rows are means ± SE among three tree species for each life form. Different lower and upper case letters indicate significant differences (p< 0.05) among root orders within each species and between life forms, respectively.

For morphological and tissue chemical traits, absorptive root SRL and tissue N concentrations were consistently and markedly reduced, while the C:N ratio increased, with increasing root order for each species regardless of angiosperm and gymnosperm species (**Table 3**). However, the intraspecific variations of tissue densities and tissue C concentrations were more dependent on life forms (*i.e.*, angiosperms and gymnosperms) or specific tree species (**Table 3**). For example, as root order increased, tissue density increased in angiosperms but remained unchanged in gymnosperms, whereas C concentration was unchanged in *F. mandschurica* and *Pinus koraiensis* (*p* > 0.05), while it increased in the other four species (**Table 3**).

**Table 3:** The mean and standard error of morphological and tissue chemical traits for the first three absorptive root orders in three angiosperms and three gymnosperms tree species (*n* = 6).

Traits	Root order	*df*	*Juglans mandshurica*	*Fraxinus mandschurica*	*Phellodendron amuense*	*Picea koraiensis*	*Larix* *gmelinii*	*Pinus koraiensis*	*df*	Angiosperms	Gymnosperms
Morphological traits											
Specific root	1	5	13.3 ± 0.8 a	14.7 ± 0.3 a	5.8 ± 0.5 a	7.7 ± 0.1 a	4.5 ± 0.5 a	2.4 ± 0.03 a	1	11.3 ± 2.4 A	4.9 ± 1.4 B
length (cm/mg)	2	5	10.4 ± 1.7 a	7.1 ± 0.6 b	3.6 ± 0.3 b	4.2 ± 0.4 b	3.1 ± 0.2 b	1.8 ± 0.1 b	1	7.0 ± 2.1 A	3.0 ± 0.7 B
	3	5	5.9 ± 0.4 b	4.2 ± 0.3 c	1.7 ± 0.2 c	2.6 ± 0.3 c	2.2 ± 0.1 b	1.6 ± 0.1 c	1	3.9 ± 1.1 A	2.1 ± 0.3 B
Tissue density	1	5	129.5 ± 3.7 b	121.6 ± 3.5 c	98.3 ± 9.0 b	187.2 ± 2.9 a	255.9 ± 14.1 a	264.0 ± 6.8 a	1	116.5 ± 11.1 B	235.7 ± 23.8 A
(mg/cm^3^)	2	5	150.6 ± 15.1 b	174.3 ± 2.0 b	111.5 ± 5.7 b	181.4 ± 13.4 a	249.7 ± 8.5 a	254.3 ± 14.3 a	1	145.5 ± 19.8 B	228.5 ± 25.7 A
	3	5	210.5 ± 0.4 a	223.3 ± 2.6 a	170.4 ± 3.4 a	226.9 ± 1.6 a	236.7 ± 0.3 a	241.8 ± 1.3 a	1	201.422.4 B	235.1 ± 22.6 A
Tissue chemical traits											
N concentration	1	5	27.5 ± 0.2 a	27.5 ± 0.4 a	26.8 ± 0.3 a	21.7 ± 0.6 a	21.7 ± 0.8 a	19.3 ± 0.6 a	1	27.3 ± 0.3 A	20.9 ± 0.9 B
(g/kg)	2	5	22.1 ± 0.6 b	24.1 ± 0.3 b	24.2 ± 0.2 b	19.6 ± 0.5 b	18.9 ± 0.5 b	17.6 ± 0.1 b	1	23.5 ± 0.7 A	18.7 ± 0.6 B
	3	5	22.1 ± 0.7 c	20.3 ± 0.2 c	20.4 ± 0.3 c	15.8 ± 0.2 c	16.9 ± 0.3 c	16.0 ± 0.4 c	1	20.5 ± 0.3 A	16.2 ± 0.4 B
C concentration	1	5	481.0 ± 0.6 b	469.9 ± 6.0 a	474.9 ± 0.4 b	464.5 ± 1.9 b	461.6 ± 0.6 c	463.2 ± 1.2 a	1	475.3 ± 5.4 A	463.1 ± 1.9 B
(g/kg)	2	5	487.5 ± 0.6 a	473.5 ± 4.5 a	475.0 ± 0.6 b	469.5 ± 1.6 b	466.1 ± 0.7 b	461.2 ± 1.1 a	1	478.7 ± 5.1 A	465.6 ± 2.6 B
	3	5	487.4 ± 0.4 a	477.2 ± 2.6 a	482.3 ± 3.4 a	478.5 ± 1.6 a	476.6 ± 0.3 a	461.9 ± 1.3 a	1	482.3 ± 4.1 A	472.3 ± 4.7 B
C:N ratio	1	5	17.5 ± 0.1 c	17.1 ± 0.1 c	17.7 ± 0.1 c	21.4 ± 0.5 c	21.4 ± 0.6 c	24.0 ± 0.4 c	1	17.4 ± 0.2 B	22.3 ± 1.0 A
	2	5	22.1 ± 0.4 b	19.7 ± 0.3 b	19.6 ± 0.1 b	24.0 ± 0.5 b	24.8 ± 0.6 b	26.3 ± 0.1 b	1	20.5 ± 0.8 B	25 ± 0.8 A
	3	5	23.4 ± 0.2 a	23.5 ± 0.2 a	23.7 ± 0.2 a	30.3 ± 0.3 a	28.2 ± 0.3 a	28.9 ± 0.5 a	1	23.5 ± 0.3 B	29.1 ± 0.7 A

For each trait, data present in the rightmost two columns are means ± SE among three tree species for each life form. Different lower and upper case letters indicate significant differences (p< 0.05) among root orders within each species and between life forms, respectively.

### 3.2 Relationships between CSRs and anatomical, morphological, and tissue chemical traits of absorptive roots

At the anatomical level, the CSR generally exhibited positive and negative relationships with cortical cell-related traits and conduit-related traits, respectively, as root orders changed in each species regardless of life forms (**Tables 4** and **5**). For conduit traits, CSRs were significantly negatively correlated with the numbers of conduits per stele (*R* ^2^ = 0.21 ~ 0.78, *p*< 0.05) and the total cross-sectional areas of conduits (*R* ^2^ = 0.29 ~ 0.72, *p*< 0.05) within each species. However, the relationship between the CSR and mean conduit diameter was significant only in four species: *F. mandschurica* (*R* ^2^ = 0.30), *P. amurense* (*R* ^2^ = 0.42), *L. gmelinii* (*R* ^2^ = 0.51), and *Picea koraiensis* (*R* ^2^ = 0.51, *p*< 0.05, **Table 4**). In contrast, the positive relationships between CSRs and cortical cell traits of mean diameter of cortical cell (only significant in two species, *R* ^2^ = 0.20 ~ 0.30), number of cortical cell layer (only significant in five species, *R* ^2^ = 0.61 ~ 0.92) and cross-sectional area of cortex (only significant in four species, *R* ^2^ = 0.33 ~ 0.86) among root orders were generally less pervasive. Overall, for each of conduit and cortical cell traits, their correlation degrees with CSR were more dependent on tree species and the specific trait rather than life forms (**Tables 4**, **5**, **Tables S1 and S2**).

**Table 4:** Group-wise regression of the ratio of unilateral cortex thickness to stele radius (CSR) and conduit traits in three angiosperms and three gymnosperms tree species.

	*Juglans mandschurica*	*Fraxinus mandschurica*	*Phellodendron amurense*	*Picea koraiensis*	*Larix* *gmelinii*	*Pinus koraiensis*	All species
CSR & Mean conduit diameter (MCD)							
Adj *R* ^2^	< 0.01	0.30	0.42	0.51	0.51	0.14	0.18
*p*	0.64	**0.01**	**< 0.01**	**< 0.01**	**< 0.01**	0.07	**< 0.01**
Standard error	0.55	0.32	0.12	0.06	0.05	0.03	0.04
*df*	17	17	17	17	17	17	107
CSR & Number of conduits per stele (NCS)							
Adj *R* ^2^	0.72	0.23	0.57	0.63	0.78	0.21	< 0.01
*p*	**< 0.01**	**0.03**	**< 0.01**	**< 0.01**	**< 0.01**	**0.03**	0.36
Standard error	< 0.01	< 0.01	< 0.01	< 0.01	< 0.01	< 0.01	< 0.01
*df*	17	17	17	17	17	17	107
CSR & Total cross-sectional area of conduits (TCAC)							
Adj *R* ^2^	0.67	0.29	0.58	0.63	0.72	0.29	0.04
*p*	**< 0.01**	**0.01**	**< 0.01**	**< 0.01**	**< 0.01**	**0.01**	**0.03**
Standard error	< 0.01	< 0.01	< 0.01	< 0.01	< 0.01	< 0.01	< 0.01
*df*	17	17	17	17	17	17	107

CSR was dependent variable (y), MCD, NCS and TCAC were independent variables (x). Significant regressions are indicated in bold type (p< 0.05). Different lower-case letters indicate significant differences (p< 0.05) in the slopes among species.

**Table 5:** Group-wise regression of the ratio of unilateral cortex thickness to stele radius (CSR) and cortical cells traits in three angiosperms and three gymnosperms tree species.

	*Juglans mandschurica*	*Fraxinus mandschurica*	*Phellodendron amurense*	*Picea koraiensis*	*Larix* *gmelinii*	*Pinus koraiensis*	All species
CSR & Mean diameter of cortical cell (MDCC)							
Adj *R* ^2^	0.30	< 0.01	0.20	0.06	< 0.01	0.17	< 0.01
*p*	**0.01**	0.72	**0.04**	0.17	0.34	0.05	0.31
Standard error	0.15	0.30	0.07	0.03	0.04	0.01	0.03
*df*	17	17	17	17	17	17	107
CSR & Number of cortical cell layer (NCCL)							
Adj *R* ^2^	0.92	0.68	< 0.01	0.64	0.87	0.61	0.82
*p*	**< 0.01**	**< 0.01**	0.58	**< 0.01**	**< 0.01**	**< 0.01**	**< 0.01**
Standard error	0.04	0.09	0.21	0.07	0.03	0.04	0.02
*df*	17	17	17	17	17	17	107
CSR & Cross-sectional area of cortex (CAC)							
Adj *R* ^2^	0.86	0.16	0.39	0.33	0.03	0.47	0.30
*p*	**< 0.01**	0.06	**< 0.01**	**< 0.01**	0.24	**< 0.01**	**< 0.01**
Standard error	< 0.01	< 0.01	< 0.01	< 0.01	< 0.01	< 0.01	< 0.01
*df*	17	17	17	17	17	17	107

CSR was dependent variable (y), MDCC, NCCL and CAC were independent variables (x). Significant regressions are indicated in bold type (p< 0.05). Different lower-case letters indicate significant differences (p< 0.05) in the slopes among species.

Among morphological and tissue chemical traits, the CSR was negatively correlated with the C:N ratio (*R* ^2^ = 0.47 ~ 0.91, *p*< 0.01, **Table 7**) but significantly positively correlated with SRL (*R* ^2^ = 0.36 ~ 0.80, *p*< 0.05, **Table 6**) and tissue N concentration (*R* ^2^ = 0.48 ~ 0.93, *p*< 0.01, **Table 7**) as root orders changed within each species. For these correlations, the correlation degrees were more dependent on tree species rather than life forms (**Tables 6** and **7**). By contrast, relationships between the CSR and root diameter, tissue density, and tissue C concentration were generally negative, but significance depended on tree species (**Tables 6**, **7**, **Tables S3 and S4**).

**Table 6:** Group-wise regression of the ratio of unilateral cortex thickness to stele radius (CSR) and root diameter, specific root length and tissue density in three angiosperms and three gymnosperms tree species.

	*Juglans mandschurica*	*Fraxinus mandschurica*	*Phellodendron amurense*	*Picea koraiensis*	*Larix* *gmelinii*	*Pinus koraiensis*	All species
CSR & Root diameter (RD)							
Adj *R* ^2^	0.10	< 0.01	0.55	0.68	0.49	0.17	< 0.01
*p*	0.11	0.69	**< 0.01**	**< 0.01**	**< 0.01**	0.05	0.31
Standard error	< 0.01	< 0.01	< 0.01	< 0.01	< 0.01	< 0.01	< 0.01
*df*	17	17	17	17	17	17	107
CSR & Specific root length (SRL)							
Adj *R* ^2^	0.47	0.47	0.47	0.80	0.36	0.44	0.26
*p*	**< 0.01**	**< 0.01**	**< 0.01**	**< 0.01**	**< 0.01**	**< 0.01**	**< 0.01**
Standard error	0.05	0.04	0.06	0.02	0.05	0.07	0.02
*df*	17	17	17	17	17	17	107
CSR & Tissue density (TD)							
Adj *R* ^2^	0.40	0.68	0.18	0.03	0.11	< 0.01	0.54
*p*	**< 0.01**	**< 0.01**	**0.04**	0.24	0.10	0.96	**< 0.01**
Standard error	< 0.01	< 0.01	< 0.01	< 0.01	< 0.01	< 0.01	< 0.01
*df*	17	17	17	17	17	17	107

CSR was dependent variable (y), RD, SRL and TD were independent variables (x). Significant regressions are indicated in bold type (p< 0.05). Different lower-case letters indicate significant differences (p< 0.05) in the slopes among species.

**Table 7:** Group-wise regression of the ratio of unilateral cortex thickness to stele radius (CSR) and N concentration, C concentration and C:N ratio in three angiosperms and three gymnosperms tree species.

	*Juglans mandschurica*	*Fraxinus mandschurica*	*Phellodendron amurense*	*Picea koraiensis*	*Larix* *gmelinii*	*Pinus koraiensis*	All species
CSR & N concentration (NC)							
Adj *R* ^2^	0.93	0.63	0.75	0.70	0.70	0.48	0.73
*p*	**< 0.01**	**< 0.01**	**< 0.01**	**< 0.01**	**< 0.01**	**< 0.01**	**< 0.01**
Standard error	0.03	0.06	0.03	0.02	0.02	0.02	0.02
*df*	17	17	17	17	17	17	107
CSR & C concentration (CC)							
Adj *R* ^2^	0.78	0.36	0.05	0.50	0.79	< 0.01	< 0.01
*p*	**< 0.01**	**< 0.01**	0.19	**< 0.01**	**< 0.01**	0.61	0.29
Standard error	0.04	0.02	0.03	0.01	0.01	0.01	0.01
*df*	17	17	17	17	17	17	107
CSR & C:N ratio (CNR)							
Adj *R* ^2^	0.91	0.72	0.69	0.70	0.77	0.47	0.65
*p*	**< 0.01**	**< 0.01**	**< 0.01**	**< 0.01**	**< 0.01**	**< 0.01**	**< 0.01**
Standard error	0.03	0.05	0.04	0.01	0.01	0.01	0.02
*df*	17	17	17	17	17	17	107

CSR was dependent variable (y), NC, CC and CNR were independent variables (x). Significant regressions are indicated in bold type (p< 0.05). Different lower-case letters indicate significant differences (p< 0.05) in the slopes among species.

At the interspecific level, CSRs were also tightly correlated with anatomical, morphological and tissue chemical traits in absorptive roots among six tree species (**Figure S1**). Specifically, for tissue chemical traits, in line with the intraspecific correlations, CSRs were more associated with N concentration and C:N ratio as compared with C concentration at the interspecific level. However, for correlations between CSRs with anatomical and morphological traits, the interspecific results differed from those of intraspecific results. For example, CSR was more closely related to cortical cell layer than to number of conduits per stele, and more closely related to tissue density than to root diameter and SRL among six tree species (**Figure S1**).

### 3.3 Differences in CSR and its associations with anatomical, morphological, and tissue chemical traits among root orders of absorptive roots between angiosperm and gymnosperm species

Listed based on the root diameter of absorptive roots, from small to large, the studied tree species were *J. mandshurica*, *F. mandschurica* and *P. amurense* in angiosperms and *Picea koraiensis*, *L. gmelinii* and *Pinus koraiensis* in gymnosperms (**Figure 1**). For root diameter, there were no significant differences between angiosperms and gymnosperms but significant differences among tree species within each group (**Table 8**). Similarly, stele radius and cortex thickness, as well as their ratios to root radius, were significantly affected by tree species within each life form (*i.e.*, angiosperms and gymnosperms), but in this case, the life forms were significantly different from each other (**Table 8**). Compared with three angiosperm species, gymnosperm species had obviously higher stele radii and greater proportions of stele radius to root radius, but lower cortex thicknesses and smaller proportions of cortex thickness to root radius, though *K*_s_ was similar in both groups (**Figures 1** and **2**, **Table 8**). Such distribution of cortex thickness and stele radius induced a dramatically higher CSR in three angiosperm species than gymnosperm species (**Figure 3A**). In three angiosperm species, the CSR was significantly higher in thick- than in thin-root species (CSR value in angiosperm species: 1.4–2.6), whereas in three gymnosperm species, the interspecific differences were not significant (CSR value in gymnosperm species: 0.4–0.7, *p* > 0.05, **Figure 3A**).

**Table 8:** ANOVA results (*p* value) of life form (*i.e*., angiosperms and gymnosperms), root order and their interaction effect, as well as tree species, root order and their interaction effect for each life form on absorptive root diameter and anatomical traits.

Source of variation	*df*	Root diameter	Cortex thickness	Stele radius	CSR	Cort : RD ratio	Stele : RD ratio	*K*s
Life form	1	0.33	**<0.01**	**<0.01**	**<0.01**	**<0.01**	**<0.01**	0.19
Root order	2	**0.03**	**<0.01**	0.08	**<0.01**	**<0.01**	**<0.01**	**<0.01**
Life form × Root order	2	0.67	0.25	0.79	**<0.01**	0.11	**0.05**	0.28
Angiosperms								
Tree species	2	**<0.01**	**<0.01**	**<0.01**	**<0.01**	**<0.01**	**<0.01**	**<0.01**
Root order	2	**<0.01**	**<0.01**	**<0.01**	**<0.01**	**<0.01**	**<0.01**	**<0.01**
Tree species ×Root order	4	**<0.01**	0.14	**<0.01**	**0.04**	**<0.01**	**<0.01**	**<0.01**
Gymnosperms								
Tree species	2	**<0.01**	**<0.01**	**<0.01**	**<0.01**	**0.01**	**<0.01**	**<0.01**
Root order	2	**<0.01**	**<0.01**	**<0.01**	**<0.01**	**<0.01**	**<0.01**	**<0.01**
Tree species ×Root order	4	**0.02**	**0.01**	0.45	**<0.01**	**<0.01**	**0.01**	0.31

CSR, Ratio of unilateral cortex thickness to stele radius; Cort : RD ratio, Ratios of bilateral cortex thickness to root diameter, Stel : RD ratio: Ratios of stele diameter to root diameter; Ks, the specific conductivity.

Comparing cortical cell and conduit traits, three angiosperm species had significantly greater numbers of cortical cell layers and cross-sectional areas of the cortex but significantly lower mean conduit diameters than three gymnosperm species in each root order (**Tables 1** and **2**). But differences between life forms in the number of conduits per stele, the total cross-sectional area of conduits, and mean diameter of cortical cells were more dependent on root order (**Tables 1** and **2**). Regarding the phenotypic traits, absorptive roots of angiosperm species had significantly higher SRL, tissue N, and C concentrations but lower tissue density and C:N ratios (**Table 3**).

At the intraspecific level, the relationships between CSRs and anatomical, morphological, and tissue chemical traits were more dependent on tree species than which life form the species belonged to (**Table 4**, **5**, **6** and **7**). The only clear difference between angiosperms and gymnosperms was the relationship between the CSR and tissue density, which was significant in all three angiosperm species (*R* ^2^ = 0.18 ~ 0.68, *p*< 0.05) but not in any gymnosperm species (*p* > 0.05, **Table 6**). However, when comparing the regression slopes of CSRs and anatomical, morphological, and tissue chemical traits among tree species, the difference was more dramatic among three angiosperm species (mainly between *P. amurense* and both of *J. mandshurica* and *F. mandschurica*, except for number of cortical cell layer and cross-sectional area of cortex) rather than among gymnosperm species (except for mean diameter of cortical cell and C concentration between *L. gmelinii* and *Pinus koraiensis*, **Tables S1-4**).

## 4 Discussion

### 4.1 Intraspecific variations of CSRs indicate the relative strength of absorptive root absorption and transportation, but are not governed by the compensatory effect

Our study found that the variation of CSRs at the intraspecific level could directly indicate the strength of absorption and transportation in absorptive roots. In the current study, the CSR declined with increasing root order within each species regardless of life forms. According to the definition of the CSR, the ratio of cortex thickness to stele radius, this decline should indicate that absorption decreased while vertical transportation increased from lower to higher root orders (Kong et al., 2017; Wang et al., 2018). Comparing these results to previous studies is restricted by a lack of CSR variation in previous studies. Only Guo et al. (2008b), which studied the ratio of stele-to-root diameter in the absorptive roots of 23 temperate tree species, is comparable, also finding consistent results that stele proportion generally increased with root order. The intraspecific variations of CSR should be attributable to root development because root development progresses with increasing root order, resulting in the emergence of secondary growth in higher-order roots (Esau, 1977; Guo et al., 2008b; Gu et al., 2014). Accordingly, a stronger stele and stronger conduits develop in roots while the cortex remains relatively vulnerable, and more serious cumulative damage to the cortex in higher- than in lower-order roots (Guo et al., 2008b; Jia et al., 2013) could explain the decrease of the CSR with the increase of root order.

Intraspecific variations of anatomical, morphological, and tissue chemical traits of absorptive roots provide further supporting evidence for the efficacy of CSR as a functional indicator at the intraspecific level. In our study, as absorptive root order increased, root diameter, tissue density, C concentration, the C:N ratio, and the number and diameter of conduits increased, but SRL, N concentration, and the diameter and number of cortical cell layers all declined. These results are consistent with those of previous studies (Pregitzer et al., 2002; Guo et al., 2008b; Jia et al., 2013; Wang et al., 2022). The above functional trait changes suggest that, as root order increased, absorptive root vertical transportation enhanced, C investment and lifespan increased, but absorption and metabolism weakened (Pregitzer et al., 2002; Comas et al., 2012; Jia et al., 2013; Gu et al., 2014). These changes in function are consistent with the functional indications of the CSR, confirming its ability to indicate the relative strength of absorption and transportation of absorptive roots remains effective at the intraspecific level.

What is believed to be an important driver of the CSR, the compensatory effect, described among tree species of angiosperms, should not exist at the intraspecific level, be the species angiosperm or gymnosperm, because the CSR variation of absorptive roots at the intraspecific level in our study contrasts with those previously reported at the interspecific level (Wang et al., 2018). Furthermore, the compensatory effect at the interspecific level was attributed to an optimally functional balance between nutrient absorption and transportation (Kong et al., 2017), so, as we proposed in the first hypothesis, we may infer that this optimally functional balance was absent at the intraspecific level. Instead, CSR could indicate the effect of the level of root development on absorption and transportation. In other words, higher CSR values in the first- than in the third-order roots suggest that lower-order roots should be more focused on the enhancement of absorption but higher-order roots more on transportation.

### 4.2 Intraspecific relationships between the CSR and anatomical, morphological, and tissue chemical traits of absorptive roots could indicate their resource acquisition strategies

Among the phenotypic traits of morphology and tissue chemistry, the CSR of absorptive roots was only significantly correlated to SRL, tissue N concentration, and the C:N ratio for each species regardless of life forms, supporting our second hypothesis. These functional traits are sensitive to environmental changes (*e.g.*, soil N availability), reacting to maximise the effective uptake of water and nutrients (Ostonen et al., 2007; Terzaghi et al., 2013; Wang et al., 2018; Wang et al., 2022). Changes to these sensitive phenotypic traits could result in corresponding changes in the cortex and stele, and thus their ratio, due to the determining role of these two tissues in absorption and transportation, respectively. Such synchronous changes may result in the close relationships seen between CSR and these three phenotypic traits in absorptive roots. Thus, our study suggests a close and sensitive mechanism underlying the association between root structure and function. However, root diameter is directly influenced by the cortex thickness and stele radius, but surprisingly, the relationship between root diameter and CSR within species was weaker and not always statistically significant. This is because cortex thickness and stele radius varied disproportionately with root diameter among root orders, particularly in the uncorrelated species (**Figures 1** and **2**), showing that cortex and stele development follows an allometric relationship at the intraspecific level, *i.e.*, a different nonlinear relationship between the development rates of the cortex and stele exists for each species. Although there are allometric relationships at both the intra- and inter-specific levels, the reasons behind them should be different. The former is caused by root development or other factors influencing the cortex tissue (Kong et al., 2017) whereas the latter is related to the compensatory effect.

For anatomical traits of absorptive roots, CSR was more tightly associated with the abundance-related traits of cortical cells and conduits than the diameter-related traits. This is because the intraspecific differences between root orders of abundance-related traits (as a percent of the species mean, differences were 17.5%~49.4% for cortical cells and 28.2%~90.2% for conduits) were obviously larger than those of diameter-related traits (mean differences were 1.3~10.0% for cortical cells and 4.2%~12.9% for conduits). Our results likely mean that the absorption and transportation of absorptive roots are mainly modulated *via* variations in the number of cortical cells and conduits but not their diameter. Chimungu et al., 2014; Chimungu et al., 2014b) suggested that, compared with the mean diameter of cortical cells, the number of cortical cell layers was a more important determinant of root metabolic costs, such as root respiration, growth, and ion uptake, because larger cortical cells have a higher ratio of vacuolar to cytoplasmic volume and thus could reduce metabolic costs of absorptive roots (Lynch, 2013). For conduit traits, compared with a greater number of conduits, larger conduit diameters could enhance vertical transport efficiency to a greater extent (Tyree and Ewers, 1991) but was more vulnerable to embolisms (Ewers et al., 1990; Hacke et al., 2000). The more prevalent, closer relationship between CSR and abundance-related traits than diameter-related traits of cortical cells and conduits probably infers that the developments of absorption and transportation of absorptive roots is more tightly regulated by the changes in the demand of metabolic cost and hydraulic safety than the efficiency of resource acquisition.

Furthermore, regarding anatomical traits, we also note that the CSR was more closely related to transport traits (*i.e.*, conduit traits) than absorptive traits (*i.e.*, cortical cell traits) in absorptive roots for both angiosperm and gymnosperm species. This may be related to the different dependencies of root function on cortical cells and conduits because, in addition to root cortex, absorption also relies on mycorrhizas (Koide, 1991; Brundrett, 2002; Eissenstat et al., 2015) whereas transportation only relies on root conduits. We thus speculate that conduits are more important in determining the extent of absorptive root development than cortical cells at the intraspecific level. However, owing to the design of our study that combining the first three order roots as measurement unit, we were not able to disentangle the effect of root order on the correlations of CSR with vital functional traits. This is a topic for future research to investigate the explicit influence of root orders on functional linkages of absorptive roots.

### 4.3 Differences in the CSR and its trait associations between these angiosperm and gymnosperm species could indicate their differences in resource acquisition strategies

In our study, different interspecific variations of CSR between three angiosperm and three gymnosperm species could reflect a stronger compensatory effect (via the cortex thickness) acting in these angiosperm species. Absorptive root CSRs significantly increased from thin- to thick-rooted species in three angiosperm species, while they changed little among the gymnosperm species (**Figure 3A**). In angiosperms, the compensatory effect is the allometric relationship proposed in previous studies that, as absorptive root thickness increases, cortex thickness must increase at a greater rate than stele radius to compensate for an inherent lag of absorption behind transportation (Valverde-Barrantes et al., 2016; Kong et al., 2017; Kong et al., 2019). This theory is confirmed by our results in the three angiosperm species: *K*s and CSR both increased significantly from thin- to thick-rooted species, suggesting an obvious improvement in transportation and the existence of the compensatory effect, respectively. However, in the three gymnosperm species, CSR remained constant among species despite a significantly increasing *K*s from thin- to thick-rooted species. This suggests weak compensation *via* the regulation of cortex for the lag of absorption in three gymnosperm species. But this does not imply that the compensatory effect observed in angiosperms does not exist in these gymnosperm species because, despite weaker volumetric flow rates in gymnosperms than in angiosperms (Guo et al., 2008b), a more pronounced enhancement of transportation than absorption from thin- to thick-root species was consistent between life forms (Feild & Arens, 2007; Kong et al., 2017). Driven by functional balance, to compensate for nutrient absorption lag, the gymnosperm species in current study should be more reliant on mycorrhizas. Indeed, previous studies confirmed that the mycorrhizal colonization of absorptive roots was higher in tree species of gymnosperms than that of angiosperms (Guo et al., 2008b).

In addition, the angiosperms’ mean CSR, averaged across the three tree species, was significantly higher than that of gymnosperms, possibly reflecting differences in resource acquisition strategies between these species. Few studies have focused on the comparison of CSR between angiosperms and gymnosperms, only Guo et al. (2008b), which studied the ratio of stele to root diameter, has studied this, finding similar results to ours. Such differences between these angiosperm and gymnosperm species should be related to their distinct features at the anatomical level. On one hand, compared with vessels in angiosperms, the relatively lower volumetric flow rate of tracheids in the absorptive roots of gymnosperms requires a higher proportion of stele in the root (as well as a higher mean conduit diameter, **Table 1**) to achieve a comparable transport capacity (Sperry et al., 2006; Guo et al., 2008b, **Table 8**). On the other hand, a thinner cortex, seen as fewer numbers of cortical cell layers and lower cross-sectional areas of the cortex in our study (**Table 2**), is beneficial for water and nutrient uptake efficiencies due to a lower resistance of water transport across the cortex (Rieger and Litvin, 1999; Wells and Eissenstat, 2003; Gu et al., 2014) and thus contribute to a better adaptation of these gymnosperm species to harsh environments (Sperry et al., 2006; Piper et al., 2019). However, note that the comparison of the differences in root functional traits between angiosperms and gymnosperms in our study was based on a limited number of tree species, more research using large samples is needed to understand the extent of these differences more comprehensively.

As we proposed in the third hypothesis, when comparing the relationships between CSR and anatomical, morphological, and tissue chemical traits, there was poor concordance between angiosperms and gymnosperms in our study, which was probably caused by the different features of nutrient acquisition by mycorrhizas. Few previous researchers have involved these symbioses in absorptive root studies, but the differing associations of absorptive root morphological with tissue chemical traits between arbuscular (AM) and ectomycorrhizal (EM) tree species reported by Kong et al. (2019) may shed light on our results. These authors suggested that the opposing associations of functional traits were due to different mycorrhizal traits between the AM and EM tree species, such as mycorrhizal types and hyphal foraging precision. In our study, the species of angiosperms and gymnosperms are infected by AM and EM, respectively (Guo et al., 2008b). Because of the different features of nutrient acquisition of these mycorrhizal types, the associations between the CSR and anatomical, morphological, and tissue chemical traits in absorptive roots may not be comparable between these species. However, compared with three angiosperm species, the regression slopes of CSR and vital functional traits were more similar among three gymnosperm species, suggesting that the intraspecific functional correlations were more stable in these gymnosperm species. This difference of the life forms probably gives rise to the different patterns of intraspecific and interspecific correlations of functional traits, as the effect of life form was not disentangled in the interspecific correlations.

## 5 Conclusion

This study investigated the efficacy of the cortex-to-stele ratio (CSR) of absorptive roots as a functional indicator at both the intra- and interspecific levels in six temperate tree species. For each species, CSR declined with increasing root orders—combined with similar functional indications from various anatomical, morphological, and tissue chemical traits—this suggests that CSR is able to indicate the relative strength of absorption and transportation of absorptive roots at the intraspecific level. This in turn suggests that root development, rather than the compensatory effect known from interspecific studies, drives changes in CSR at the intraspecific level. Comparing angiosperm and gymnosperm species, CSR was significantly different among species of angiosperms but displayed little change among species of gymnosperms, further suggesting that the compensatory effect *via* the regulation of cortex thickness was much stronger in angiosperm species in our study. Furthermore, CSR could indicate strategies in resource acquisition at both the intra- and interspecific levels. Among anatomical, morphological, and tissue chemical traits, significant associations of CSR were observed only with SRL, N concentration, the C:N ratio, and the number of and total cross-sectional area of conduits in each species, suggesting a close and sensitive mechanism underlying the association between root structure and function within species. In addition, three angiosperm species, overall, had a higher mean CSR than three gymnosperm species, but the relationship between CSR and other functional traits was not comparable between life forms, which we attribute to distinct features of anatomical structure and nutrient acquisition *via* different mycorrhiza. However, these gymnosperm species have a more stable intraspecific functional linkage than three angiosperm species. These results showed that the absorption of three gymnosperm species may rely more on efficiency in soil nutrient acquisition than three angiosperm species to maintain the balance with transportation, but within species of both life forms, the absorption and transportation were more tightly regulated by root development. Our study on the variation and association of the CSR of absorptive roots reflects complex strategies in resource acquisition for trees in terrestrial ecosystems and provides information for the scientific basis of forestry management.

## Data availability statement

The original contributions presented in the study are included in the article/**Supplementary Material**. Further inquiries can be directed to the corresponding author.

## Author contributions

WW and SW conceived and designed this research project. XL, YR, WW, and YD performed the data analysis. WW, SW, YW, and ZL completed the field work. XL, YR and SW performed the laboratory experiment. WW and YD supervised this work. XL, YD, YR, SW, YW, ZL, and WW contributed to the revisions and comments concerning the manuscript. All authors contributed to the article and approved the submitted version.

## Supplementary material

Supplementary Figure 1 Relationships among the ratio of unilateral cortex thickness to stele radius (CSR) and root anatomical, morphological and tissue chemical traits among three angiosperm and three gymnosperm tree species (*n* = 6). CSR, cortical thickness to stele radius ratio; RD, root diameter; SRL, specific root length; TD, tissue density; NCPS, number of conduits per stele; MCD, mean conduit diameter; TCAC, total cross-sectional area of conduits; MDCC, mean diameter of cortical cell; NCCL, number of cortical cell layer; CAC, Cross-sectional area of cortex; NC, N concentration; CC, C concentration; CNR, C:N ratio.
