# Supplementary material for: New insights into the cortex-to-stele ratio show it to effectively indicate inter- and intraspecific function in the absorptive roots of temperate trees
Source: Front Plant Sci. 2023 Jan 20;14:1061503. doi: 10.3389/fpls.2023.1061503 (PMC9895863; doi:10.3389/fpls.2023.1061503)
Supplement: Supplementary file 1 [file Table_1.docx]

**Table S1** Standardized major axis analysis of the ratio of unilateral cortex thickness to stele radius (CSR) and conduit traits in three angiosperms and three gymnosperms tree species (*n* = 18).

|  | *Juglans mandschurica* | *Fraxinus mandschurica* | *Phellodendron amurense* | *Picea koraiensis* | *Larix*  *gmelinii* | *Pinus koraiensis* |
| --- | --- | --- | --- | --- | --- | --- |
| ***CSR & Mean conduit diameter (MCD)*** | | | | | | |
| Slope | -11.37 a | -6.99 ab | -1.95 d | -6.78 ab | -4.86 bc | -5.30 bc |
| Intercept | 8.63 | 5.37 | 2.22 | 5.73 | 4.41 | 5.31 |
| *R*^2^ | < 0.01 | 0.26 | 0.44 | 0.57 | 0.52 | 0.20 |
| *p* | 0.85 | **0.03** | **< 0.01** | **< 0.01** | **< 0.01** | 0.06 |
| ***CSR & Number of conduits per stele (NCS)*** | | | | | | |
| Slope | -1.21 a | -1.00 a | -0.30 b | -1.32 a | -1.01 a | -1.66 a |
| Intercept | 1.72 | 1.66 | 0.91 | 1.30 | 1.21 | 2.05 |
| *R*^2^ | 0.74 | 0.30 | 0.87 | 0.84 | 0.80 | 0.25 |
| *p* | **< 0.01** | **0.02** | **< 0.01** | **< 0.01** | **< 0.01** | **0.03** |
| ***CSR & Total cross-sectional area of conduits (TCAC)*** | | | | | | |
| Slope | -1.23 a | -0.86 ab | -0.24 c | -0.99 ab | -0.74 b | -1.17 ab |
| Intercept | 3.50 | 2.65 | 1.24 | 2.57 | 2.16 | 3.74 |
| *R*^2^ | 0.79 | 0.36 | 0.83 | 0.83 | 0.77 | 0.31 |
| *p* | **< 0.01** | **< 0.01** | **< 0.01** | **< 0.01** | **< 0.01** | **0.02** |

Note: CSR was dependent variable (y), MCD, NCS and TCAC were independent variables (x). All data were log-transformed prior to analysis. Significant regressions are indicated in bold type (*p* < 0.05). Different lower-case letters indicate significant differences (*p* < 0.05) in the slopes among species.

**Table S2** Standardized major axis analysis of the ratio of unilateral cortex thickness to stele radius (CSR) and cortical cells traits in three angiosperms and three gymnosperms tree species (*n* = 18).

|  | *Juglans mandschurica* | *Fraxinus mandschurica* | *Phellodendron amurense* | *Picea koraiensis* | *Larix*  *gmelinii* | *Pinus koraiensis* |
| --- | --- | --- | --- | --- | --- | --- |
| ***CSR & Mean diameter of cortical cell (MDCC)*** | | | | | | |
| Slope | 7.36 ab | 12.16 a | -2.55 cd | 2.65 cd | 4.44 bc | 2.19 d |
| Intercept | -7.84 | -12.94 | 3.80 | -3.52 | -5.69 | -3.22 |
| *R*^2^ | 0.31 | 0.02 | 0.24 | 0.23 | 0.06 | 0.30 |
| *p* | **0.02** | 0.58 | **0.04** | **0.04** | 0.33 | **0.02** |
| ***CSR & Number of cortical cell layer (NCCL)*** | | | | | | |
| Slope | 2.18 a | 2.13 a | 2.55 a | 2.19 a | 1.83 a | 1.98 a |
| Intercept | -1.36 | -1.34 | -1.90 | -1.05 | -1.01 | -1.29 |
| *R*^2^ | 0.95 | 0.90 | 0.03 | 0.66 | 0.91 | 0.66 |
| *p* | **< 0.01** | **< 0.01** | 0.50 | **< 0.01** | **< 0.01** | **< 0.01** |
| ***CSR & Cross-sectional area of cortex (CAC)*** | | | | | | |
| Slope | 1.30 a | 1.38 a | -1.24 a | 2.08 a | 1.93 a | 1.50 a |
| Intercept | -5.53 | -5.93 | 6.85 | -9.07 | -8.80 | -7.37 |
| *R*^2^ | 0.86 | 0.66 | 0.47 | 0.41 | 0.11 | 0.52 |
| *p* | **< 0.01** | **< 0.01** | **< 0.01** | **< 0.01** | 0.19 | **< 0.01** |

Note: CSR was dependent variable (y), MDCC, NCCL and CAC were independent variables (x). All data were log-transformed prior to analysis. Significant regressions are indicated in bold type (*p* < 0.05). Different lower-case letters indicate significant differences (*p* < 0.05) in the slopes among species.

**Table S3** Standardized major axis analysis of the ratio of unilateral cortex thickness to stele radius (CSR) and root diameter, specific root length and tissue density in three angiosperms and three gymnosperms tree species (*n* = 18).

|  | *Juglans mandschurica* | *Fraxinus mandschurica* | *Phellodendron amurense* | *Picea koraiensis* | *Larix*  *gmelinii* | *Pinus koraiensis* |
| --- | --- | --- | --- | --- | --- | --- |
| ***CSR & Root diameter (RD)*** | | | | | | |
| Slope | 6.94 a | 5.89 ab | -2.02 c | -4.05 b | -3.93 b | -3.99 ab |
| Intercept | -16.35 | -13.86 | 5.83 | 9.43 | 9.68 | 9.97 |
| *R*^2^ | 0.29 | 0.02 | 0.65 | 0.80 | 0.52 | 0.23 |
| *p* | **0.02** | 0.56 | **< 0.01** | **< 0.01** | **< 0.01** | **0.04** |
| ***CSR & Specific root length (SRL)*** | | | | | | |
| Slope | 0.40 c | 0.58 bc | 2.27 a | 0.65 b | 0.67 b | 0.49 bc |
| Intercept | 0.98 | 0.79 | -0.42 | 0.88 | 0.63 | 0.51 |
| *R*^2^ | 0.68 | 0.31 | 0.57 | 0.72 | 0.41 | 0.44 |
| *p* | **< 0.01** | **0.02** | **< 0.01** | **< 0.01** | **< 0.01** | **< 0.01** |
| ***CSR & Tissue density (TD)*** | | | | | | |
| Slope | -3.72 a | -3.60 a | -0.86 b | -3.36 a | 5.48 a | 4.02 a |
| Intercept | 8.12 | 8.15 | 2.20 | 7.31 | -13.32 | -10.12 |
| *R*^2^ | 0.54 | 0.61 | 0.29 | 0.06 | 0.13 | 0.01 |
| *p* | **< 0.01** | **< 0.01** | **0.02** | 0.32 | 0.14 | 0.71 |

Note: CSR was dependent variable (y), RD, SRL and TD were independent variables (x). All data were log-transformed prior to analysis. Significant regressions are indicated in bold type (*p* < 0.05). Different lower-case letters indicate significant differences (*p* < 0.05) in the slopes among species.

**Table S4** Standardized major axis analysis of the ratio of unilateral cortex thickness to stele radius (CSR) and N concentration, C concentration and C:N ratio in three angiosperms and three gymnosperms tree species (*n* = 18).

|  | *Juglans mandschurica* | *Fraxinus mandschurica* | *Phellodendron amurense* | *Picea koraiensis* | *Larix*  *gmelinii* | *Pinus koraiensis* |
| --- | --- | --- | --- | --- | --- | --- |
| ***CSR & N concentration (NC)*** | | | | | | |
| Slope | 8.20 a | 7.43 ab | 2.14 d | 5.58 bc | 4.45 abc | 5.30 abc |
| Intercept | -11.27 | -10.07 | -2.53 | -7.51 | -5.90 | -7.07 |
| *R*^2^ | 0.78 | 0.56 | 0.78 | 0.80 | 0.67 | 0.50 |
| *p* | **< 0.01** | **< 0.01** | **< 0.01** | **< 0.01** | **< 0.01** | **< 0.01** |
| ***CSR & C concentration (CC)*** | | | | | | |
| Slope | -147.95 a | -41.08 bc | -21.01 d | -51.94 bc | -36.23 cd | 72.39 b |
| Intercept | 397.33 | 110.05 | 56.68 | 138.44 | 96.55 | -193.37 |
| *R*^2^ | 0.60 | 0.34 | 0.11 | 0.61 | 0.77 | < 0.01 |
| *p* | **< 0.01** | **0.01** | 0.17 | **< 0.01** | **< 0.01** | 0.84 |
| ***CSR & C:N ratio (CNR)*** | | | | | | |
| Slope | -7.84 a | -7.02 ab | -2.02 d | -5.09 bc | -4.02 c | -5.37 abc |
| Intercept | 10.29 | 9.27 | 3.04 | 6.73 | 5.38 | 7.15 |
| *R*^2^ | 0.78 | 0.64 | 0.76 | 0.79 | 0.69 | 0.51 |
| *p* | **< 0.01** | **< 0.01** | **< 0.01** | **< 0.01** | **< 0.01** | **< 0.01** |

Note: CSR was dependent variable (y), NC, CC and CNR were independent variables (x). All data were log-transformed prior to analysis. Significant regressions are indicated in bold type (*p* < 0.05). Different lower-case letters indicate significant differences (*p* < 0.05) in the slopes among species.
