# Supplementary figures and images for: New insights into the cortex-to-stele ratio show it to effectively indicate inter- and intraspecific function in the absorptive roots of temperate trees
Source: Front Plant Sci. 2023 Jan 20;14:1061503. doi: 10.3389/fpls.2023.1061503 (PMC9895863; doi:10.3389/fpls.2023.1061503)

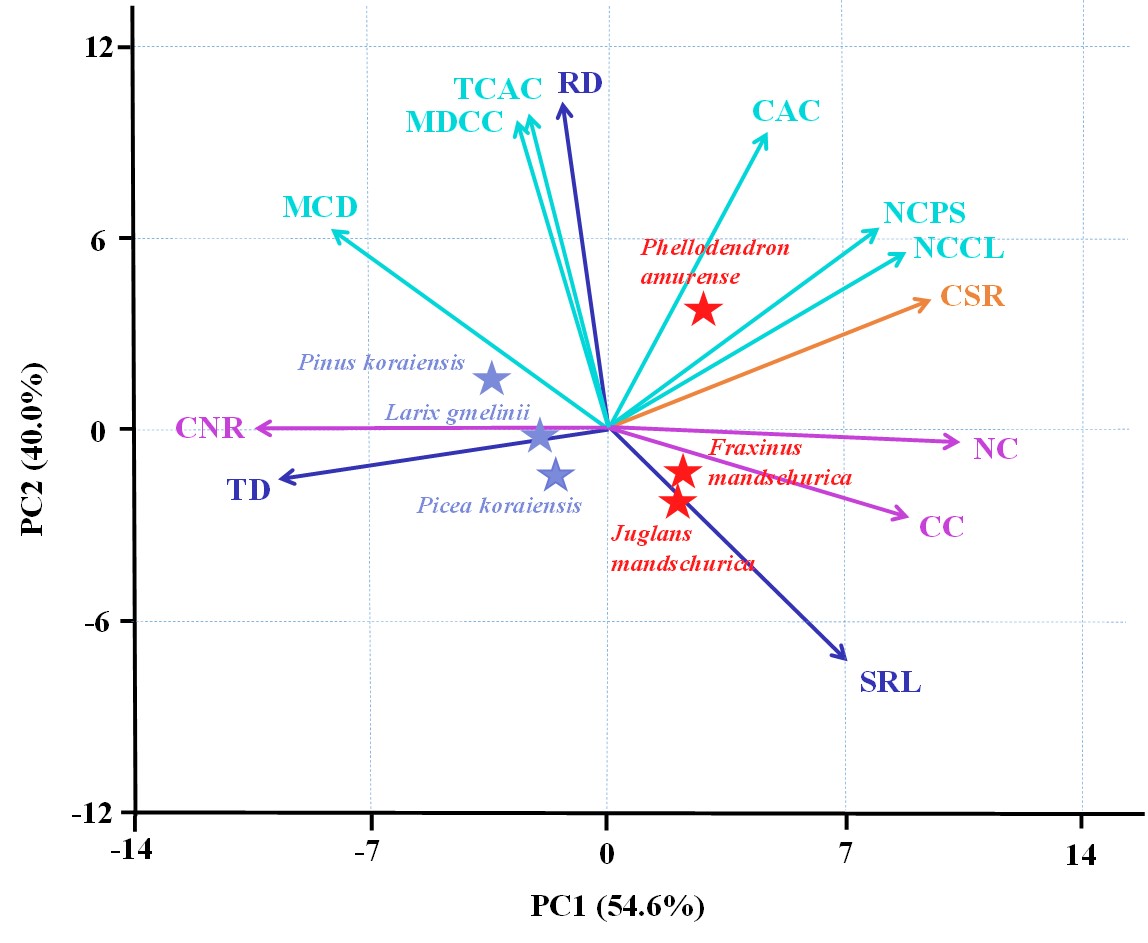

Supplement: Supplementary Figure 1 — Relationships among the ratio of unilateral cortex thickness to stele radius (CSR) and root anatomical, morphological and tissue chemical traits among three angiosperm and three gymnosperm tree species (n = 6). CSR, cortical thickness to stele radius ratio; RD, root diameter; SRL, specific root length; TD, tissue density; NCPS, number of conduits per stele; MCD, mean conduit diameter; TCAC, total cross-sectional area of conduits; MDCC, mean diameter of cortical cell; NCCL, number of cortical cell layer; CAC, Cross-sectional area of cortex; NC, N concentration; CC, C concentration; CNR, C:N ratio. [file Image_1.jpg]
